# Supplementary material for: Introns control stochastic allele expression bias
Source: Nat Commun. 2021 Nov 11;12:6527. doi: 10.1038/s41467-021-26798-4 (PMC8585970; doi:10.1038/s41467-021-26798-4)
Supplement: Supplementary file 1 — Supplementary Information [file 41467_2021_26798_MOESM1_ESM.pdf]

Supplementary Information for

Introns Control Stochastic Allele Bias

Bryan Sands, Soo Yun and Alexander R. Mendenhall.

Correspondence to: alexworm@uw.edu

This PDF file includes:

Supplementary Note 1: Statistical Analyses…..…………………………...…Pages 2-6
Supplementary Figs.1-2........………………………………………………...Pages 7-10
Supplementary Tables 1-5……………………………………………..Pages 11-20
Caption for Source Data 1………..…..…………………………………………...Page 21
Caption for Supplementary Data 1…...……………………………………...Page 21

Other Supplementary Materials for this manuscript include the following:

Source Data 1
Supplementary Data 1

Supplementary Text

Results of Statistical Analyses

Below, we detail the results of each statistical analysis of our data on intrinsic noise levels.

==

Mann Whitney U tests:

HSP-90 muscle cells chr II

Mann-Whitney Rank Sum Test

Group	N	Missing	Median	25%	75%
no i hsp90	180	0	0.0545	0.0174	0.161
i hsp90	180	0	0.00391	0.000639	0.0168

Mann-Whitney U Statistic= 7345.000

T = 41345.000 n(small)= 180 n(big)= 180 (P = <0.001)

The difference in the median values between the two groups is greater than would be expected by chance; there is a statistically significant difference (P = <0.001)

MYO-3 muscle cells

Mann-Whitney Rank Sum Test

Group	N	Missing	Median	25%	75%
myo-3 no introns	180	0	0.00555	0.00155	0.0176
myo-3 with introns	180	0	0.00162	0.000276	0.00428

Mann-Whitney U Statistic= 9657.000

T = 39033.000 n(small)= 180 n(big)= 180 (P = <0.001)

The difference in the median values between the two groups is greater than would be expected by chance; there is a statistically significant difference (P = <0.001)

HSP-90 Muscle Cells Chr V.

Mann-Whitney Rank Sum Test

Group	N	Missing	Median	25%	75%
No INtron Muscle	240	0	0.117	0.0182	0.393
Muscle Introns	240	0	0.0679	0.0224	0.187

Mann-Whitney U Statistic= 25781.000

T = 60739.000 n(small)= 240 n(big)= 240 (P = 0.047)

The difference in the median values between the two groups is greater than would be expected by chance; there is a statistically significant difference (P = 0.047)

HSP-16.2 Intestine Cells

Mann-Whitney Rank Sum Test

Group	N	Missing	Median	25%	75%
hsp162 i noise	280	0	0.00312	0.000637	0.0127
hsp162 no i noise	277	0	0.00817	0.00200	0.0262

Mann-Whitney U Statistic= 29129.000

T = 86934.000 n(small)= 277 n(big)= 280 (P = <0.001)

The difference in the median values between the two groups is greater than would be expected by chance; there is a statistically significant difference (P = <0.001)

VIT-2 Intestine Cells

Mann-Whitney Rank Sum Test

Group	N	Missing	Median	25%	75%
vit2 i noise	279	0	0.000948	0.000327	0.00433
vit2 no i noise	280	0	0.00514	0.000803	0.0219

Mann-Whitney U Statistic= 24801.000

T = 63861.000 n(small)= 279 n(big)= 280 (P = <0.001)

The difference in the median values between the two groups is greater than would be expected by chance; there is a statistically significant difference (P = <0.001)

HSP-90 Intestine Cells Chr. V.

Mann-Whitney Rank Sum Test

Group	N	Missing	Median	25%	75%
No Introns	559	0	0.0270	0.00612	0.0989
Introns	560	0	0.0193	0.00399	0.0620

Mann-Whitney U Statistic= 138979.000

T = 330581.000 n(small)= 559 n(big)= 560 (P = 0.001)

The difference in the median values between the two groups is greater than would be expected by chance; there is a statistically significant difference (P = 0.001)

Tissue:

Mann-Whitney Rank Sum Test

Group	N	Missing	Median	25%	75%
intestine	1674	0	0.0158	0.00289	0.0609
muscle	840	0	0.0470	0.00651	0.199

Mann-Whitney U Statistic= 523485.500

T = 1235894.500 n(small)= 840 n(big)= 1674 (P = <0.001)

The difference in the median values between the two groups is greater than would be expected by chance; there is a statistically significant difference (P = <0.001)

Locus:

Mann-Whitney Rank Sum Test

Group	N	Missing	Median	25%	75%
II	915	0	0.00894	0.00173	0.0488
V	1599	0	0.0331	0.00663	0.118

Mann-Whitney U Statistic= 534578.500

T = 953648.500 n(small)= 915 n(big)= 1599 (P = <0.001)

The difference in the median values between the two groups is greater than would be expected by chance; there is a statistically significant difference (P = <0.001)

5' vs 3' introns

Normality Test (Shapiro-Wilk) Failed (P < 0.050)

Mann-Whitney Rank Sum Test

Group	N	Missing	Median	25%	75%
5' hsp90	280	0	0.00244	0.000639	0.00814
3' hsp90	280	0	0.00957	0.00191	0.0311

Mann-Whitney U Statistic= 25363.000

T = 64703.000 n(small)= 280 n(big)= 280 (P = <0.001)

The difference in the median values between the two groups is greater than would be expected by chance; there is a statistically significant difference (P = <0.001)

HSP-90 T2A

Mann-Whitney Rank Sum Test

Normality Test (Shapiro-Wilk) Failed (P < 0.050)

Group	N	Missing	Median	25%	75%
hsp90 no i	252	0	0.00665	0.00114	0.0208
hsp90 i	263	0	0.00149	0.000383	0.00566

Mann-Whitney U Statistic= 20457.000

T = 77697.000 n(small)= 252 n(big)= 263 (P = <0.001)

The difference in the median values between the two groups is greater than would be expected by chance; there is a statistically significant difference (P = <0.001)

MTL-2 T2A

Mann-Whitney Rank Sum Test

Normality Test (Shapiro-Wilk) Failed (P < 0.050)

Group	N	Missing	Median	25%	75%
mtl2 no i	178	0	0.00422	0.000992	0.0114
mtl2 i	179	0	0.00211	0.000472	0.0108

Mann-Whitney U Statistic= 14037.000

T = 33756.000 n(small)= 178 n(big)= 179 (P = 0.052)

The difference in the median values between the two groups is not great enough to exclude the possibility that the difference is due to random sampling variability; there is not a statistically significant difference (P = 0.052)

==

ANOVAs

HSP-90 Alleles on Chr 2 in Intestine Cells:

Normality Test (Shapiro-Wilk) Failed (P < 0.050)

Kruskal-Wallis One Way Analysis of Variance on Ranks

Group	N	Missing	Median	25%	75%
no i hsp90	278	0	0.0259	0.00542	0.0642
i hsp90	277	0	0.00237	0.000642	0.00755
nat i hsp90	279	0	0.00333	0.000556	0.0117

H = 160.470 with 2 degrees of freedom. (P = <0.001)

The differences in the median values among the treatment groups are greater than would be expected by chance; there is a statistically significant difference (P = <0.001)

To isolate the group or groups that differ from the others use a multiple comparison procedure.

All Pairwise Multiple Comparison Procedures (Dunn's Method) :

Comparison	Diff of Ranks	Q	P<0.05
no i hsp90 vs i hsp90	234.865	11.484	Yes
no i hsp90 vs nat i hsp90	211.743	10.372	Yes
nat i hsp90 vs i hsp90	23.122	1.132	No

Normality Test (Shapiro-Wilk) Failed (P < 0.050)

Group	N	Missing	Median	25%	75%
no i hsp90	278	0	0.0259	0.00542	0.0642
i hsp90	277	0	0.00237	0.000642	0.00755
nat i hsp90	279	0	0.00333	0.000556	0.0117

H = 160.470 with 2 degrees of freedom. (P = <0.001)

The differences in the median values among the treatment groups are greater than would be expected by chance; there is a statistically significant difference (P = <0.001)

To isolate the group or groups that differ from the others use a multiple comparison procedure.

All Pairwise Multiple Comparison Procedures (Dunn's Method) :

Comparison	Diff of Ranks	Q	P<0.01
no i hsp90 vs i hsp90	234.865	11.484	Yes
no i hsp90 vs nat i hsp90	211.743	10.372	Yes
nat i hsp90 vs i hsp90	23.122	1.132	No

Note: The multiple comparisons on ranks do not include an adjustment for ties.

Promoters:

Normality Test (Shapiro-Wilk) Failed (P < 0.050)

**Group**	**N**	**Missing**	**Median**	**25%**	**75%**
hsp-90	555	0	0.00682	0.00138	0.0338
hsp-16.2	557	0	0.00492	0.00107	0.0180
vit-2	559	0	0.00216	0.000445	0.0101

H = 73.473 with 2 degrees of freedom. (P = <0.001)

The differences in the median values among the treatment groups are greater than would be expected by chance; there is a statistically significant difference (P = <0.001)

To isolate the group or groups that differ from the others use a multiple comparison procedure.

All Pairwise Multiple Comparison Procedures (Dunn's Method) :

**Comparison**	**Diff of Ranks**	**Q**	**P<0.05**
hsp-90 vs vit-2	243.515	8.422	Yes
hsp-90 vs hsp-16.2	82.281	2.843	Yes
hsp-16.2 vs vit-2	161.235	5.581	Yes

Note: The multiple comparisons on ranks do not include an adjustment for ties.

Normality Test (Shapiro-Wilk) Failed (P < 0.050)

**Group**	**N**	**Missing**	**Median**	**25%**	**75%**
hsp-90	555	0	0.00682	0.00138	0.0338
hsp-16.2	557	0	0.00492	0.00107	0.0180
vit-2	559	0	0.00216	0.000445	0.0101

H = 73.473 with 2 degrees of freedom. (P = <0.001)

The differences in the median values among the treatment groups are greater than would be expected by chance; there is a statistically significant difference (P = <0.001)

To isolate the group or groups that differ from the others use a multiple comparison procedure.

All Pairwise Multiple Comparison Procedures (Dunn's Method) :

**Comparison**	**Diff of Ranks**	**Q**	**P<0.01**
hsp-90 vs vit-2	243.515	8.422	Yes
hsp-90 vs hsp-16.2	82.281	2.843	No
hsp-16.2 vs vit-2	161.235	5.581	Yes

Note: The multiple comparisons on ranks do not include an adjustment for ties.

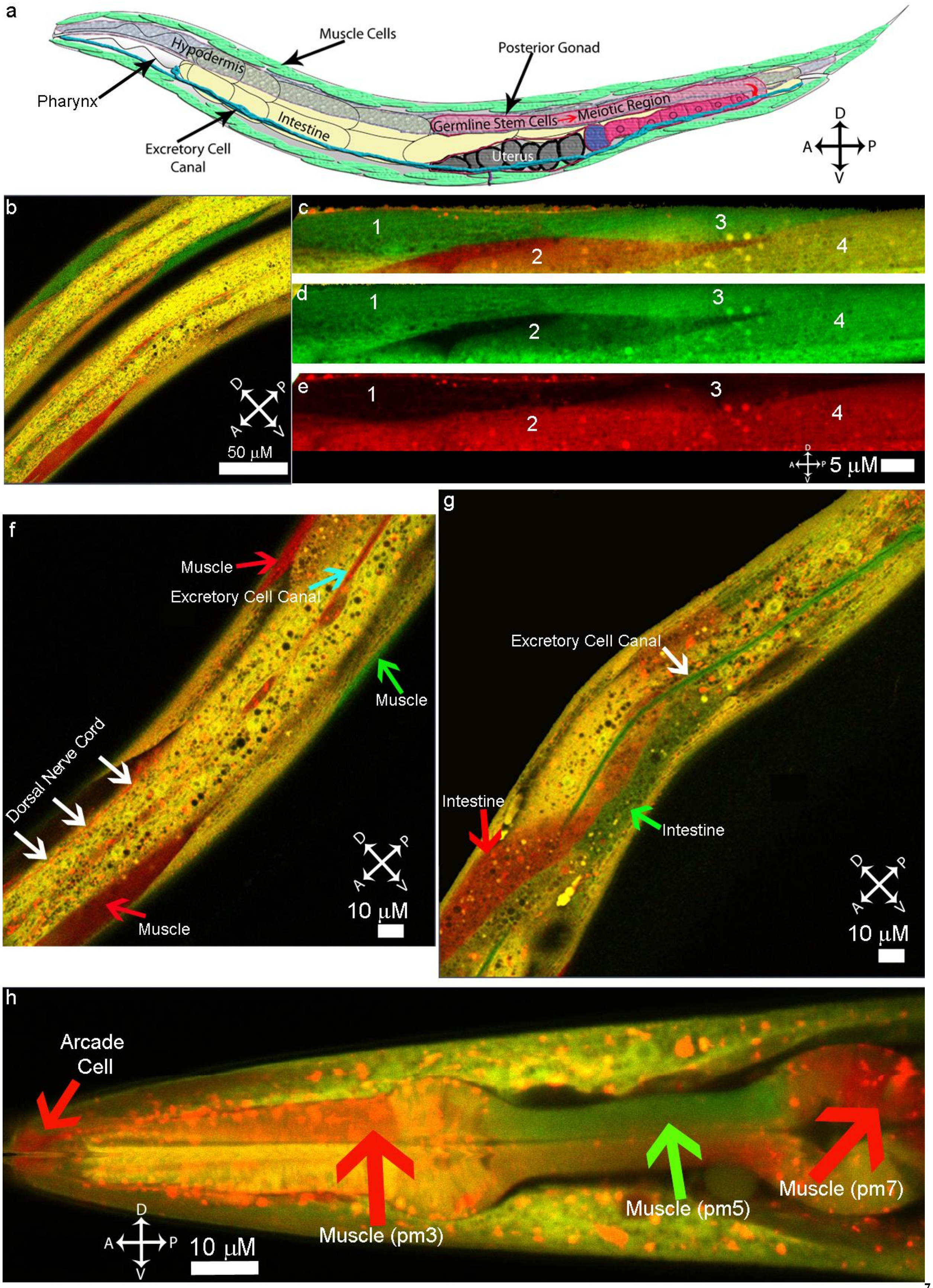
a
Muscle Cells
Posterior Gonad
Hypodermis
Pharynx
Germline Stem Cells
Meiotic Region
Intestine
Excretory Cell Canal
Uterus
D
A
P
V
b
50 µM
c
1
2
3
4
d
1
2
3
4
e
1
2
3
4
5 µM
f
Muscle
Excretory Cell Canal
Muscle
Dorsal Nerve Cord
Muscle
10 µM
g
Excretory Cell Canal
Intestine
Intestine
10 µM
h
Arcade Cell
Muscle (pm3)
Muscle (pm5)
Muscle (pm7)
10 µM

Supplementary Figure 1. A survey of stochastic allele bias in the somatic tissues of *C. elegans*. a) shows a cartoon diagram of *C. elegans* body. The cartoon details the positions of relevant tissues in C. elegans body. **b-h** show point-scanning confocal micrographs of animals expressing red and green alleles with introns, controlled by the *hsp-90* promoter. **b)** shows a merged red/green signal micrograph of two animals' anterior sections with a visibly yellow hypodermis, and distinct green- and red-allele-biased striated body wall muscles visible against the black background and yellow hypodermal tissues. **c-e)** show micrographs of striated body wall muscles, labeled 1-4, with **c)** showing a red/green merged image, **d)** showing the green channel (note the absence of signal for cell 2), and **e)** showing the red channel (note the absence of signal for cells 1&3). Scale bar in **e** applies to **c,d**. **f)** shows a merged red/green signal micrograph of an animal with stochastic allele bias visible in a nerve cord, striated muscles, and the excretory cell canal. **g)** shows a merged red/green signal micrograph of an animal with stochastic allele bias visible in intestine cells and the excretory cell canal. **h)** shows a merged red/green signal micrograph of an animal with stochastic allele bias visible in the arcade cells and in the smooth muscles of the pharynx. Images with clear bias and good cell positioning were selected form four independent experiments capturing z-stack image sets of ten, two-day-old adult animals in each experiment.

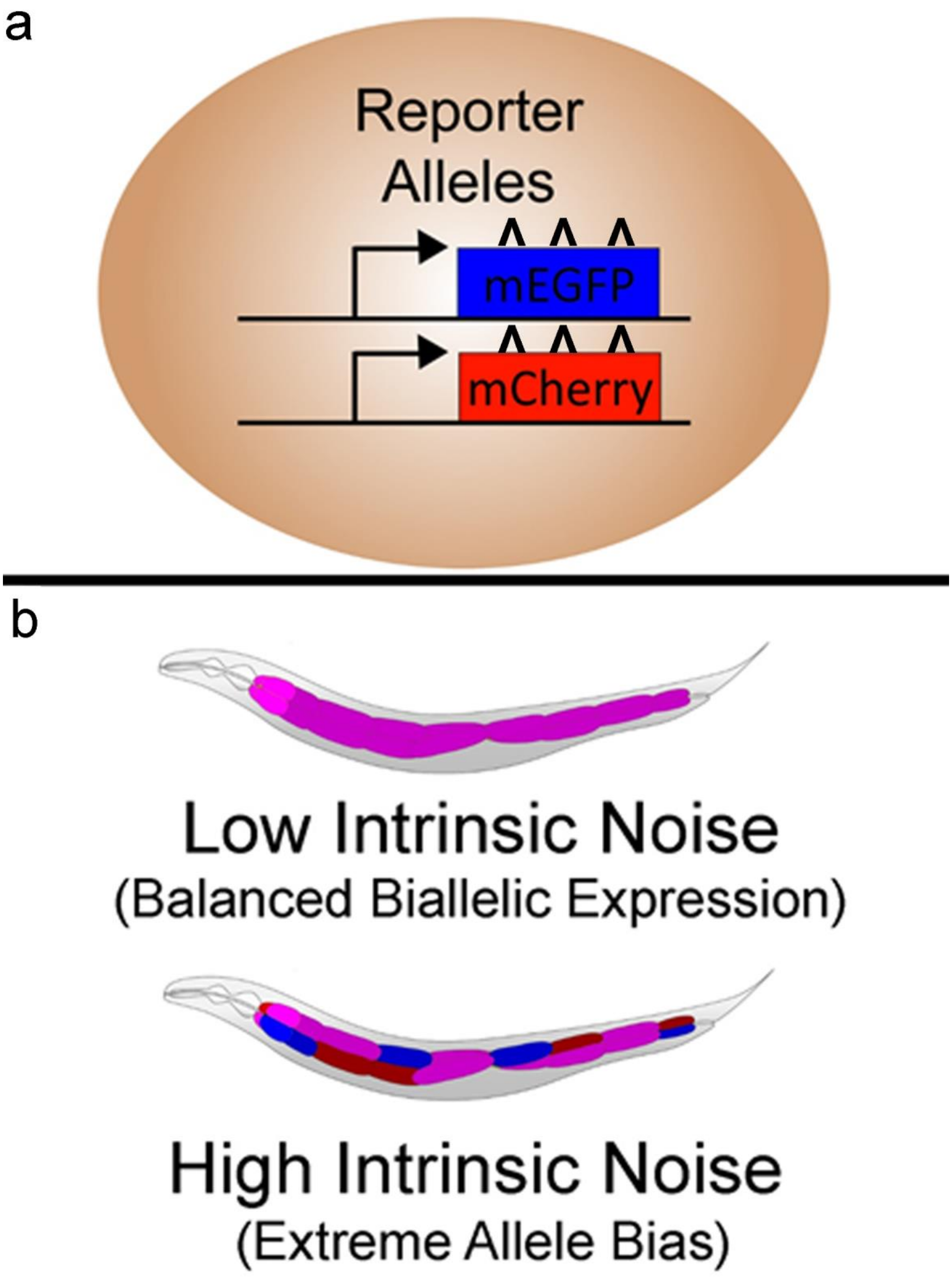
a
Reporter
Alleles
mEGFP
mCherry
b
Low Intrinsic Noise
(Balanced Biallelic Expression)
High Intrinsic Noise
(Extreme Allele Bias)

Supplementary Figure 2. Colorblind Corrected Condensed Experimental Schematics. a) shows a cartoon detailing that cells contain two otherwise identical copies of alleles encoding two spectrally distinct fluorescent proteins; here mEGFP is false colored blue to obviate red/green color blindness. **b)** shows cartoon examples of what high and low intrinsic noise would look like among the twenty cells comprising the worm intestine tissue. The top panel shows low intrinsic noise, which appear as purple cells (or somewhat pink), because purple is the balanced blend of red and blue. The lower panel shows high intrinsic noise, which appears as an array of randomly biased and balanced cells; some cells appear purple, and some cells are mostly, or all, red or blue. We will happily recolor any raw microscopic images and send representative or desired images of particular z slices to any interested colorblind individuals.

Cell Type	Locus	Description of Alleles	Number of Cells	Number of Experiments	Median Intrinsic Noise	Spearman R^2	*P* for Intrinsic Noise
Striated Muscle	II	*hsp-90* promoter, XFPs without introns	180	3	0.0545	3.1%	< 0.001
Striated Muscle	II	*hsp-90* promoter, XFPs with 3 synthetic introns	180	3	0.00391	30.6%	
Striated Muscle	V	*hsp-90* promoter, XFPs without introns	240	4	0.117	0.1%	< 0.05
Striated Muscle	V	*hsp-90* promoter, XFPs with 3 synthetic introns	240	4	0.0679	7.1%	
Striated Muscle	I	*myo-3* promoter, XFPs without introns	180	3	0.00555	83.4%	< 0.001
Striated Muscle	I	*hsp-90* promoter, XFPs with 3 synthetic introns	180	3	0.00162	95.3%	
Intestine	II	*hsp-90* promoter, XFPs without introns	278	4	0.0259	56.1%	< 0.01
Intestine	II	*hsp-90* promoter, XFPs with 3 synthetic introns	277	4	0.00237	90.1%	

Intestine	II	*hsp-90* promoter, XFPs with 2 *hsp-90,* or 3 synthetic, introns	279	4	0.00333	80.1%	< 0.01
Intestine	V	*hsp-90* promoter, XFPs without introns	559	8	0.027	47.2%	0.001
Intestine	V	*hsp-90* promoter, XFPs with 3 synthetic introns	560	8	0.0193	61.8%	
Intestine	II	*hsp-16.2* promoter, XFPs without introns	277	4	0.00817	67.9%	< 0.001
Intestine	II	*hsp-16.2* promoter, XFPs with 3 synthetic introns	280	4	0.00312	87.6%	
Intestine	II	*vit-2* promoter, XFPs without introns	280	4	0.00514	87.0%	< 0.001
Intestine	II	*vit-2* promoter, XFPs with 3 synthetic introns	279	4	0.00095	96.8%	
Intestine	II	*hsp-90* promoter, XFPs with a synthetic 3'-intron	280	4	0.00957	62.6%	< 0.001
Intestine	II	*hsp-90* promoter,	280	4	0.00244	87.0%	

		XFPs with a synthetic 5'-intron					
Intestine	II	full *hsp-90* promoter and coding sequence without *hsp-90* introns	252	4	0.00665	53.7%	< 0.001
Intestine	II	full *hsp-90* promoter and coding sequence with all *hsp-90*introns	263	4	0.00149	92.5%	
Intestine	II	full *mtl-2* promoter and coding sequence without *mtl-2* intron	178	3	0.00422	93.1%	> 0.05
Intestine	II	full *mtl-2* promoter and coding sequence with *mtl-2* intron	179	3	0.00211	94.3%	

Supplementary Table 1: Statistical details for comparrisons of allele bias between sets of intron-bearing and intronless alleles.

Strain Name	Genotype
ARM136	*hutSi2631[unc-119(+), P_{hsp-90}::mcherry::T_{unc-54}, II:8420158]*
ARM134	*hutSi2651[unc-119(+), P_{hsp-90}::megfp::T_{unc-54}, II:8420158]*
ARM133	*hutSi2661[unc-119(+), P_{hsp-90}:: megfp w/ 3 synthetic introns::T_{unc-54}, II:8420158]*
ARM210	*wamSi210[unc-119(+), P_{hsp-90}:: megfp w/ 5'intron::T_{unc-54}, II:8420158]*
ARM211	*wamSi210[unc-119(+), P_{hsp-90}:: megfp w/ 3'intron::T_{unc-54}, II:8420158]*
ARM212	*wamSi212[unc-119(+), P_{hsp-90}::mCherry w/ 5'intron::T_{unc-54}, II:8420158]*
ARM213	*wamSi213[unc-119(+), P_{hsp-90}::mcherry w/ 3'intron::T_{unc-54}, II:8420158]*
ARM137	*wamSi137[unc-119(+), P_{hsp-90}::mcherry w/2 hsp-90 introns::T_{unc-54}, II:8420158]*
ARM135	*hutSi2642[unc-119(+), P_{hsp-90}::mcherry w/3 synthetic introns::T_{unc-54}, II:8420158]*
ARM147	*hutSi2511[unc-119(+), P_{vit-2}::mcherry ::T_{unc-54}, II:8420158]*
ARM148	*hutSi2581[unc-119(+), P_{vit-2}::mcherry w/ 3 synthetic introns::T_{unc-54}, II:8420158]*
ARM145	*hutSi2611[unc-119(+), P_{vit-2}::megfp ::T_{unc-54}, II:8420158]*
ARM146	*hutSi2621[unc-119(+), P_{vit-2}::megfp w/ 3 synthetic introns::T_{unc-54}, II:8420158]*
ARM139	*hutSi2552[unc-119(+), $P_{hsp-16.2}$::mcherry ::T_{unc-54}, II:8420158]*
ARM140	*hutSi2561[unc-119(+), $P_{hsp-16.2}$::mcherry w/ 3 synthetic introns::T_{unc-54}, II:8420158]*
ARM138	*hutSi2591[unc-119(+), $P_{hsp-16.2}$::megfp ::T_{unc-54}, II:8420158]*
ARM141	*hutSi2601[unc-119(+), $P_{hsp-16.2}$::megfp w/ 3 synthetic introns::T_{unc-54}, II:8420158]*
ARM287	*wamSi287[unc-119(+), P_{hsp-90}::megfp ::$T_{unc-54,}$ V:8643273]*
ARM291	*wamSi291[unc-119(+), P_{hsp-90}::megfp w/3 synthetic introns::$T_{unc-54,}$ V:8643273]*
ARM290	*wamSi290[unc-119(+), P_{hsp-90}::mcherry ::$T_{unc-54,}$ V:8643273]*
ARM284	*wamSi284[unc-119(+), P_{hsp-90}::mcherry w/3 synthetic introns::$T_{unc-54,}$ V:8643273]*
ARM276	*wamSi276[unc-119(+), P_{myo-3}::megfp ::$T_{unc-54,}$ I:2851000]*
ARM273	*wamSi273[unc-119(+), P_{myo-3}::megfp w/3 synthetic introns::$T_{unc-54,}$ I:2851000]*

ARM278	*wamSi278[unc-119(+), P_{myo-3}::mcherry ::$T_{unc-54,}$ I:2851000]*
ARM270	*wamSi270[unc-119(+), P_{myo-3}::mcherry w/3 synthetic introns::$T_{unc-54,}$ I:2851000]*
ARM263	*wamSi263[unc-119(+), P_{hsp-90}::hsp-90 natural introns::t2a:: megfp ::$T_{unc-54,}$ II:8420158]*
ARM265	*wamSi265[unc-119(+), P_{hsp-90}::hsp-90 no introns:: t2a :: megfp ::$T_{unc-54,}$ II:8420158]*
ARM268	*wamSi268[unc-119(+), P_{hsp-90}::hsp-90 natural introns:: t2a :: mcherry ::$T_{unc-54,}$ II:8420158]*
ARM260	*wamSi260[unc-119(+), P_{hsp-90}::hsp-90 no introns:: t2a :: mcherry ::$T_{unc-54,}$ II:8420158]*
ARM237	*wamSi237[unc-119(+), P_{mtl-2}::mtl-2 natural introns:: t2a :: megfp ::$T_{mtl-2,}$ II:8420158]*
ARM239	*wamSi239[unc-119(+), P_{mtl-2}::mtl-2 no introns:: t2a :: megfp ::$T_{mtl-2,}$ II:8420158]*
ARM229	*wamSi229[unc-119(+), P_{mtl-2}::mtl-2 natural introns:: t2a :: mcherry ::$T_{mtl-2,}$ II:8420158]*
ARM234	*wamSi234[unc-119(+), P_{mtl-2}::mtl-2 no introns:: t2a :: mcherry ::$T_{mtl-2,}$ II:8420158]*

Supplementary Table 2. Strain names, descriptions and genotypes of reporter gene bearing *C. elegans* strains we created and used in this study.

**Crosses**	**Description of heterozygous animals**
ARM136 x ARM134♂	*hsp-90* no introns @ chr. II locus
ARM135 x ARM133♂	*hsp-90* introns @ chr. II locus
ARM212 x ARM210♂	*hsp-90* 5’ intron @ chr. II locus
ARM213 x ARM211♂	*hsp-90* 3’ intron @ chr. II locus
ARM137 x ARM133♂	*hsp-90* synthetic introns in mEGFP/natural *hsp-90* introns in mCherry @ chr. II locus
ARM147 x ARM145♂	*vit-2* no introns @ chr. II locus
ARM148 x ARM146♂	*vit-2* introns @ chr. II locus
ARM139 x ARM138♂	hsp-16.2 no introns @ chr. II locus
ARM140 x ARM141♂	hsp-16.2 introns @ chr. II locus
ARM290 x ARM287♂	hsp-90 no introns @ chr. V locus
ARM284 x ARM291♂	hsp-90 introns @ chr. V locus
ARM278 x ARM276♂	myo-3 introns @ chr. I locus
ARM270 x ARM278♂	myo-3 no introns @ chr. I locus
ARM268 x ARM263♂	HSP90-T2A natural introns @ chr. II locus
ARM260 x ARM265♂	HSP90-T2A no introns @ chr. II locus
ARM229 x ARM237♂	MTL2-T2A natural intron @ chr. II locus
ARM234 x ARM239♂	MTL2-T2A no intron @ chr. II locus

Supplementary Table 3. Initial crosses used to generate stocks of heterozygous animals that were propagated as heterozygous hermaphrodites through repeated selection and propagation of heterozygous hermaphrodites.

Synthetic intron 1	guaaguuuaaacauauauauacuaacuaacccugauuauuuaaauuuucag
Synthetic intron 2	guaaguuuaaacaguucgguacuaacuaaccauacauauuuaaauuuucag
Synthetic intron 3	uaaguuuaaacaugauuuuacuaacuaacuaaucugauuuaaauuuucag
hsp-90 intron 1	guuuguuuuuucgcuucugagucaauuuuuuaaaauaucgguuuuag
hsp-90 intron 2	guauuuuuaguuuuaauuauuuuauggcaauaauuccauuaauuucag

Supplementary Table 4. Introns used in this study in the context of mEGFP and mCherry protein coding sequence. HSP90::T2A and MTL-2 T2A experiments used the native introns, and were copied directly from Wormbase. Fluorescent protein sequences can be found in Sands et al. 2018 doi: 10.1016/j.tma.2018.01.001.

Primer	Sequence	Notes
AMO162	CTTCTCCTTTACTCATggttctggaaaaatatcaattaaca	Hsp90 for GFP A
AMO163	ttgatatttttccagaaccATGAGTAAAGGAGAAGAACTTTTCAC	Hsp-90 for GFP B
AMO341	cgacggccagtCCGTAATACGACTCACTtaAGGCCaaatgaatatttataaatatttttgatttgttgaaaaaaaaaaac	Hsp-90 for 188 for
AMO346	AGGCACGGGCGCGAGATGTCTGATGACAGCGGCCTTATTTGTATAGTTCATCCATGCCAT	Hsp-90 for 188 rev
AMO466	cgacggccagtCCGTAATACGACTCACTtaAGGCCCCCCACAAGCAATAGGAGAAGtata	vit-2 188 for
AMO467	GTCCAATCACGGTTCAGCCATGAGTAAAGGAGAAG	vit-2 GFP rev
AMO468	GTCCAATCACGGTTCAGCCATGAGTAAAGGAGAAG	GFP-vit-2 for
AMO469	GAGGCACGGGCGCGAGATGTCTGATGACAGCGGCCctaTTTGTATAGTTCATCC	GFP-un54 rev
AMO470	GTCCAATCACGGTTCAGCCatggtctcaaagggtgaag	vit-2 -cherry for
AMO471	cttcaccctttgagaccatGGCTGAACCGTGATTGGAC	vit-2 cherry rev
AMO472	GAGGCACGGGCGCGAGATGTCTGATGACAGCGGCCctacttatacaattcatccatgccacc	cherry-unc54 rev
AMO473	cgacggccagtCCGTAATACGACTCACTtaAGGCCGATCAAGAGCATTTGAATCAG	hsp16.2 188 for
AMO474	CTTCTCCTTTACTCATATGATTATAGTTTGAAGATTTC	hsp16.2 GFP rev
AMO475	GAAATCTTCAAACTATAATCATATGAGTAAAGGAGAAG	GFP for 16.2 for

AMO476	cttcttcacccctttgagaccatATGATTATAGTTTGAAGATTTC	hsp16.2 cherry rev
AMO477	GAAATCTTCAAACTATAATCATatggtctcaaagggtgaagaag	cherry for 16.2 for
AMO341	cgacggccagtCCGTAATACGACTCACTtaAGGCCaaatgaatatttataaatattttttgatttgttgaaaaaaaaaaac	hsp90 T2A A
AMO342	TTCGGCGTTCTCGGACATggttctggaaaaatatcaattaacaatcg	hsp90 T2A B
AMO343	tgatatttttccagaaccATGTCCGAGAACGCCGA	hsp90 T2A C
AMO344	ACGTCCCTCTCCAGATCCGTCGACCTCCTCCATGC	hsp90 T2A D
AMO345	CGCATGGAGGAGGTCGACGGATCTGGAGAGGGACGT	hsp90 T2A E
AMO346	AGGCACGGGCGCGAGATGTCTGATGACAGCGGCCTTATTTGTATAGTTCATCCATGCCAT	hsp90 T2A F
AMO347	AGGCACGGGCGCGAGATGTCTGATGACAGCGGCCTTACTTATACAATTCATCCATGCCAC	hsp90 T2A G
AMO348	CTGGTAGCGAATCTTGTCAAGA	hsp90 OE to remove introns
AMO349	TCTTGACAAGATTCGCTACCAGGCACTCACCGAGCCATC	hsp90 OE to remove introns
AMO350	CTTGTAGAACTCAGCATATTCTTCG	hsp90 OE to remove introns
AMO351	CGAAGAATATGCTGAGTTCTACAAGAGCTTGTCCAATGACTGGG	hsp90 OE to remove introns
AMO352	TTCATGATAGCGTGGTCTGG	hsp90 OE to remove introns

AMO353	CCAGACCACGCTATCATGAAGACACTTCGTGATC GTGTCG	hsp90 OE to remove introns
AMO388	tatttcaattgtttctcacatttcgtttg	myoskilodge outer A
AMO389	GATAATAACAAAAATAGGGGGTGGG	myoskilodge outer B
AMO390	ctagaaATGAGTAAAGGAGAAGAACTTTTC	GFP inner
AMO391	TCTGATGACAGCGGCCT	unc54 outer
AMO392	ctagaaATGGTCTCAAAGGGTGAAG	Cherry inner
AMO310	GGATCTGGAGAGGGACGT	*T2A for*
AMO315	cgacggccagtCCGTAATACGACTCACTtaAGGCCagag aatacaaaaagagacgaaaatgg	*Mtl-2 for 188 for*
AMO316	ACGTCCCTCTCCAGATCCATGAGCAGCCTGAGCA CA	*Mtl-2 T2A rev*
AMO317	tgtttcaaagggaagagtattaatttcaaaTTACTTATACAATTC ATCCATGCCACC	*Mtl-2 cherry rev*
AMO319	TACGCCAAGCTACGTAATACGACTCACTAGTGGG Caacaatttggttgatgcattaattgatttatta	*Mtl-2 rev*

Supplementary Table 5. Primers used in this study

Source Data 1. (separate file)

An excel spreadsheet detailing expression values and intrinsic noise values. X,Y coordinates corresponding to allele expression values and intrinsic noise values are listed by figure, with each figure's data in a separate sheet.

Supplementary Data 1. (separate file)

An excel spreadsheet detailing results of bioinformatic analyses. The first tab lists human protein coding genes that are intronless. The second tab shows the intronless human genes listed at the database for monoallelic gene expression (dbMAE https://mae.hms.harvard.edu/). The third and fourth sheets list the Gene Ontology term enrichment analyses for molecular functions and biological processes for human intronless genes, respectively. The fifth sheet lists intronless, protein coding genes in the *C. elegans* genome. The sixth and seventh sheets list the Gene Ontology term enrichment analyses for molecular functions and biological processes for *C. elegans* intronless genes, respectively.
