# Supplementary material for: Introns control stochastic allele expression bias
Source: Nat Commun. 2021 Nov 11;12:6527. doi: 10.1038/s41467-021-26798-4 (PMC8585970; doi:10.1038/s41467-021-26798-4)
Supplement: Supplementary file 3 — Description of Additional Supplementary Files [file 41467_2021_26798_MOESM3_ESM.pdf]

Description of Additional Supplementary Files

File Name: Supplementary Data 1
Description: Bioinformatic Analyses
